# Application of Internet of Things to Agriculture—The LQ-FieldPheno Platform: A High-Throughput Platform for Obtaining Crop Phenotypes in Field

**DOI:** 10.34133/research.0059

**Published:** 2023-03-20

**Authors:** Jiangchuan Fan, Yinglun Li, Shuan Yu, Wenbo Gou, Xinyu Guo, Chunjiang Zhao

**Affiliations:** ^1^ Beijing Key Laboratory of Digital Plant, National Engineering Research Center for Information Technology in Agriculture, Beijing 100097, China.; ^2^ Beijing Research Center for Information Technology in Agriculture, Beijing Academy of Agriculture and Forestry Sciences, Beijing 100097, China.; ^3^ China National Engineering Research Center for Information Technology in Agriculture (NERCITA), Beijing 100097, China.

## Abstract

The lack of efficient crop phenotypic measurement methods has become a bottleneck in the field of breeding and precision cultivation. However, high-throughput and accurate phenotypic measurement could accelerate the breeding and improve the existing cultivation management technology. In view of this, this paper introduces a high-throughput crop phenotype measurement platform named the LQ-FieldPheno, which was developed by China National Agricultural Information Engineering Technology Research Centre. The proposed platform represents a mobile phenotypic high-throughput automatic acquisition system based on a field track platform, which introduces the Internet of Things (IoT) into agricultural breeding. The proposed platform uses the crop phenotype multisensor central imaging unit as a core and integrates different types of equipment, including an automatic control system, upward field track, intelligent navigation vehicle, and environmental sensors. Furthermore, it combines an RGB camera, a 6-band multispectral camera, a thermal infrared camera, a 3-dimensional laser radar, and a deep camera. Special software is developed to control motions and sensors and to design run lines. Using wireless sensor networks and mobile communication wireless networks of IoT, the proposed system can obtain phenotypic information about plants in their growth period with a high-throughput, automatic, and high time sequence. Moreover, the LQ-FieldPheno has the characteristics of multiple data acquisition, vital timeliness, remarkable expansibility, high-cost performance, and flexible customization. The LQ-FieldPheno has been operated in the 2020 maize growing season, and the collected point cloud data are used to estimate the maize plant height. Compared with the traditional crop phenotypic measurement technology, the LQ-FieldPheno has the advantage of continuously and synchronously obtaining multisource phenotypic data at different growth stages and extracting different plant parameters. The proposed platform could contribute to the research of crop phenotype, remote sensing, agronomy, and related disciplines.

## Introduction

Severe changes in global climate, continuous increase in population, and reduction in arable land resources have posed many challenges to global agriculture and have brought multiple difficulties related to the resources, environment, people, and technology [1,2]. Therefore, it is urgently needed to improve agricultural production technology, accelerate agricultural development, and improve the production process, crop varieties, and quality of food. The breeding of special varieties and precise regulations in crop cultivation is important for solving the current grain problem [3]. Recently, with the continuous development of genomics and metabolomics, the mining of genes regulating important traits of crops has been dramatically accelerated [4,5]. However, the development of efficient techniques and methods for phenotypic omics has been lagging substantially and thus cannot match the massive genomic data, which greatly hinders the further development of plant breeding and becomes the bottleneck in the current breeding field [6,7]. The phenotypic data acquisition technology has a wide application range in agricultural production; for instance, it has been used in cultivation management, plant protection, water irrigation, fertilizer management, and pest control [8,9].

Plant phenotype refers to the observable morphological features generated through the interaction between the plant genotype and an environment. The research on plant phenotype has shown that the relationship between the plant phenotype and the environmental factors and plant genotypes can promote the development of plant phenomics, which has brought breakthroughs in crop science [10–12]. Plant phenotypes include traits of multiple scales from population to organs. Therefore, a fast, accurate, and efficient analysis of plant phenotypic traits has become crucial in the research and application of assistance and screening of genetic breeding and precision management of crops [13,14]. In general, plant phenotypic data have been collected using traditional manual measurement tools, but this method is time-consuming, inefficient, and has poor inaccuracy, thus limiting further development of plant phenotypic omics research. At present, there is an urgent need for plant breeders, plant pathologists, and other plant researchers who can perform fast, accurate, and error-free detection of plant character parameters to understand plant growth and development conditions and to improve crop breeding efficiency [4,12,15]. However, high-throughput platforms for crop phenotype developed abroad have been difficult to apply in China [16]. Namely, in terms of hardware, these platforms are complex, their maintenance is expensive, the replacement of pieces of equipment is difficult, the after-sale response is slow, the maintenance cycle is long, the electricity consumption is high, and user training is not always available. In addition, from the aspect of the software, the application process is tedious, and data preprocessing is necessary for images and raw spectral data. Furthermore, most indicators obtained by the analysis do not coincide with phenotypic traits concerned by breeders and agronomists. Finally, the upgrade cost of the analysis software is high.

Considering the low throughput of phenotypic acquisition of field crop population and a space–time limitation, this study integrates various sensors, including an RGB camera, a 6-band multispectral camera, a thermal infrared camera, a 3-dimensional (3D) laser radar, and a deep camera, to design a field plant high-throughput scale-based platform. The proposed platform combines different Internet of Things (IoT) technologies, such as control signal transmission, data backhaul, and working condition monitoring [17–20].

The main contributions of this study can be summarized as follows:

1. An underground base, support frame, planar scanning track, electric telescopic rod, system control module, data acquisition module, and intelligent analysis module are used to construct a high-throughput phenotyping platform. The proposed platform can realize 3D, full-automatic, full-area plant phenotype data acquisition of crop group area. In addition, various phenotype parameters, such as morphology, color, and physiology, of the crop group; a single plant; and leaves are automatically analyzed.
2. Technologies such as breeding material identification, structure measurement, physicochemical parameter analysis, and spatial–temporal dynamic monitoring are applied to the image, point cloud, and characteristic spectrum data to conduct a high-throughput analysis of large-scale phenotypes in the field. The proposed platform can analyze different phenotypic indexes, thus can provide researchers, breeders, and agronomists with a practical software tool for crop phenotypic set analysis.

The remainder of this paper is organized as follows. In Related work, the current literature is reviewed. In Materials and Methods, the proposed platform is described in detail and a phenotypic analysis algorithm is introduced. In Results, the experimental results are given. Discussion discusses the contribution and limitations of this study. Finally, Conclusions concludes the paper.

### Related work

Plant phenomics has become a crucial part of agricultural science and life science research [4,8,9]. Using high-throughput collection platform facilities for plant phenotypes, a system can obtain high time sequence plant phenotype data, and a plant phenome extensive data management system can be constructed to explore the relationship between the plant genotype phenotype and environment [9]. In recent years, variety recognition, digital breeding, and intelligent cultivation management have become the priority of plant resource analysis. Since the late 20th century, the acquisition and analysis of high-throughput, high-quality in situ plant phenotypic data have been important research directions in the field of modern agriculture and, thus, have been widely investigated. However, most of the current measurement methods of plant phenotypes still require performing plant sampling and obtaining the phenotypic parameters of plants through multi-angle vision or 3D reconstruction with depth information in a laboratory environment. Still, these methods cannot dynamically and genuinely reflect the growth of plants in a natural environment [21]. Therefore, the acquisition and analysis of high-throughput plant phenotype data based on field in situ have been highly significant. In recent research, numerous IoT technologies have been used to collect plant phenotype data to promote throughput and automation performances.

With the continuous cooperative research and development in multidisciplinary fields, including sensor technology [22], communication technology [23], plant science [24], computer science [25], and engineering science, agricultural IoT technology has made rapid progress by applying environmental sensor networks, nondestructive imaging, spectral analysis, robot technology, machine vision, and lidar technology. Agricultural IoT uses different sensor equipment and sensing technologies, as well as wireless sensor networks, mobile communication wireless networks, and the Internet, to collect data on agricultural production, product circulation, and plant body. The collected agricultural data are preprocessed, integrated, and analyzed [26]. With the rapid development of IoT technology and its introduction into agriculture, modern imaging technology, and automated industrial equipment, high-throughput techniques have been widely employed to obtain plants’ phenotypic characteristics in various growing environments using integrated imaging sensors. Numerous studies have been conducted to address the problem of low phenotypic throughout and spatial–temporal limitation of crop populations in the field, and different solutions have been proposed [16,27–29]. For instance, the PhenoWatch (https://www.zeal-quest.com/) field phenotypic analysis system is a mobile phenotypic imaging system, which was designed on the basis of the sensor-to-plant concept. In addition, 2 rack systems are used in the PhenoWatch system for measurement purposes: the field gantry version and the removable field version. The system uses a laser radar system to obtain 3D high-precision point cloud data on plants, and a variety of phenotypic parameters and information on plants are obtained through special software and algorithms. The 3D central imaging unit of the PhenoWatch for plant phenotype includes multiple measurement sensors, such as lidar, RGB, and multispectral cameras. In addition, the FieldScan is Netherlands’ 3D high-throughput plant laser scanning and measurement system. This system represents a high-throughput field plant phenotyping platform specially designed for complex field environments, which can perform the measurement under the all-weather condition in any environment. Furthermore, this system can perform full-automatic, high-throughput scale-type measurements on plants planted in natural farmland within an area of 20 m × 100 m. Moreover, it can acquire 3D multispectral point cloud data on plants and can automatically calculate phenotypic parameters, such as plant height, 3D leaf area, projected leaf area, digital biomass, leaf angle, leaf area index, light penetration depth, and leaf coverage. In addition to the phenotypic parameters, the multispectral reflection at each point of a plant can also be measured. Information, such as greenness, chlorophyll, and aging, can be obtained by analyzing a single band, whereas the normalized difference vegetation index, enhanced vegetation index, and photochemical reflectance index can be obtained by analyzing several bands. The bands measured using a multispectral camera can be customized from a list of wavelengths to meet the requirements of different application areas. The FieldScan system can be used for the measurement of a study area and individual plants. The matching environmental sensor collects ecological data of the measurement area and combines the plant growth data and environmental parameters for conducting a comprehensive analysis. The University of Nebraska–Lincoln proposed a suspended cable phenotypic platform, which covers an area of 0.4 ha and has a payload capacity of a sensor box of 30 kg. This platform integrates a 4-band multispectral camera, a thermal infrared camera, a 3D scanning lidar, and a visible near-infrared spectrometer to realize the plant parameter measurement. The sensor platform can move at a maximum speed of 2 m/s and move between any 2 positions in the imaging area within 45 s, achieving a positioning accuracy of 5 cm in the *XYZ* coordinate system. The core imaging area is divided into 128 zones, each of which has a size of 4.6 × 6.1 m and contains 6 crop rows with a row spacing of 0.76 m. The entire room is equipped with a drip irrigation system and weather stations. The remaining significant components supporting the facility operation include the subsurface drip irrigation system, electrical building, and 4 winch houses for winch operation. The platform runs every other day, collecting an average of 10 GB of data.

Among many phenotypic parameters, in situ measurement data on plant height have always been a hot research topic in crop science. The height of maize plants is an important agronomic trait related to the lodging resistance of a maize stalk and an essential index of maize phenotypic parameters. The phenotypic measurement of plant height can provide necessary data for quantitative analysis of the genotype-environment interaction effect and contribute to maize breeding [29,30]. Most of the traditional measurement methods of crop height are based on manual measurement. The vertical distance between the highest point of a plant and the soil surface is measured by placing the ruler near the plant or the infrared distance meter at the highest point of the natural growth of maize. This measurement method is substantially affected by the subjective factors of experimenters and is prone to errors. To address this problem, recent studies used a laser scanner and performed image-based fixed-point plant height extraction and RGB-D camera plant height measurement [31–33].

## Materials and Methods

### Facility overview

**Fig. 1.**
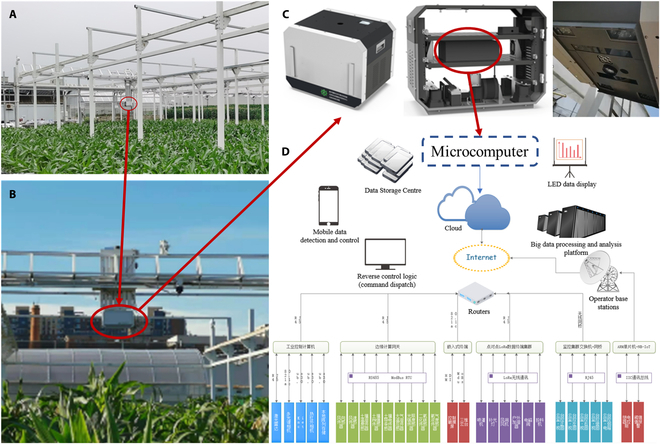
The LQ-FieldPheno field phenotyping facility: (A) picture of the facility, (B) data collection platform, (C) sensors implemented into the platform, and (D) control system of the platform.

The LQ-FieldPheno facility, which is presented in Fig. 1A, is located at the National Agricultural Information Engineering Technology Research Centre, Beijing, China (39°56′36.0″N, 116°17′11.2″E, 60 m above sea level). The portable phenotypic high-throughput automatic acquisition system based on the field track uses a crop phenotypic multisensor central imaging unit as a core and integrates different systems, including an automatic control system, an ascending field track, an intelligent navigation vehicle, and an environmental sensor. It can acquire phenotypic data of a field plant population canopy in the whole growth period in a high-throughput, full-automatic, and high time sequence manner. Moreover, this system has the characteristics of multiple acquired data, vital timeliness, robust expandability, high cost, and flexible customization. The overall efficiency of the system can reach 15 s per plot. Furthermore, the system can operate 24 h a day and continuously detect up to 5,760 zones; it processes only RGB images and can effectively measure by lidar up to 5,760 plots/day for different types of plants, including maize, wheat, rice, soybeans, vegetables, flowers, and fruit trees.

The field track platform is used for data acquisition; a depth camera and 3D lidar are employed as leading collection equipment for the maize population point cloud data. In addition, the phenotypic platform includes an industrial camera, thermal infrared camera, multispectral camera, and integrated control equipment, as shown in Fig. 1C. The running track of the phenotypic platform is 14 m long and 4 m wide and has a running speed of 5 cm/s. The platform height is 1.8 m above the maize canopy, as shown in Fig. 1B. As shown in Fig. 1D, in the control system, the height of the collection equipment can be adjusted continuously according to the current growth state of maize plants. The collection angle is perpendicular to the maize growth direction.

A powerful microcomputer (Intel NUC 11 Essential) would be installed in the data acquisition platform. The microcomputer would be responsible for data acquisition sensor calls, platform operation path planning, and data upload from other environmental sensors. In addition, a routed base station with a bandwidth of 1 GB has been set up in a nearby laboratory. The platform’s microcomputer is always in network coverage, which both reduces the latency of the control system and ensures that data are uploaded in real time. All data processing algorithms are developed in advance and stored in the cloud. Once the data have been uploaded, it can be parsed in real time via the data processing interface.

### Study area

Fourteen maize varieties, including m751, zd958, and xy335, were selected as test objects. The selected seeds were treated and sowed on 2020 June 21. One row of each variety was planted with a planting density of 60,000 seeds/ha. The data collection was conducted in the jointing stage, and the daily collection periods included 8:00 to 8:30, 13:00 to 13:30, and 18:00 to 18:30. Furthermore, the test period was from 2020 August 5 to 2020 September 30, during which the shallow temperature was 19 °C, while the extremely high temperature was 35 °C.

### Integrated sensor platform

Various plant sensors were mounted on the LQ-FieldPheno platform to capture multimodal plant traits, and they included:

• An RGB camera,
• A 6-band multispectral camera,
• A thermal infrared camera,
• A 3D laser radar, and
• A deep camera.

An NUC minicomputer with a Windows system controlled all sensor modules, collected sensors data, and stored the measurement data into the personal computer platform. Detailed information on the sensors is given in Table 1.

**Table 1.** Information on the plant sensors mounted on the LQ field sensor platform.

Sensors	Description	Advantages	Disadvantages
RGB	Exmor APS HD CMOS, Sony	Low price	Limited imaging information
	Resolution: 20.43 megapixels	Light weight	-
	ISO: I00-25600	Work flexibly	-
Multispectral	MicaSense Altum	Low price	Less bands
	Resolution: 3.2 megapixels	Easy operation	No continuous spectrum information
	Band (nm): 475, 560, 668, 717, 840, and 11,000	Easy data analysis	-
Thermal infrared	MicaSense Altum	Crop pest monitoring	High noise
	Resolution: 20.43 megapixels	Water stress response monitoring	Mixed pixel error
	Sensitivity < 50 mk	-	-
3D laser radar	VLP-16, Velodyne	High precision	High cost
	Input: 300 thousand point/s	Strong penetrability	Susceptible to environmental influences
Deep camera	Kinect V2.0, Microsoft	High speed	Difficult data processing
	Resolution: 2.0736 megapixels	Low price	Low resolution
	-	-	High noise

**Fig. 2.**
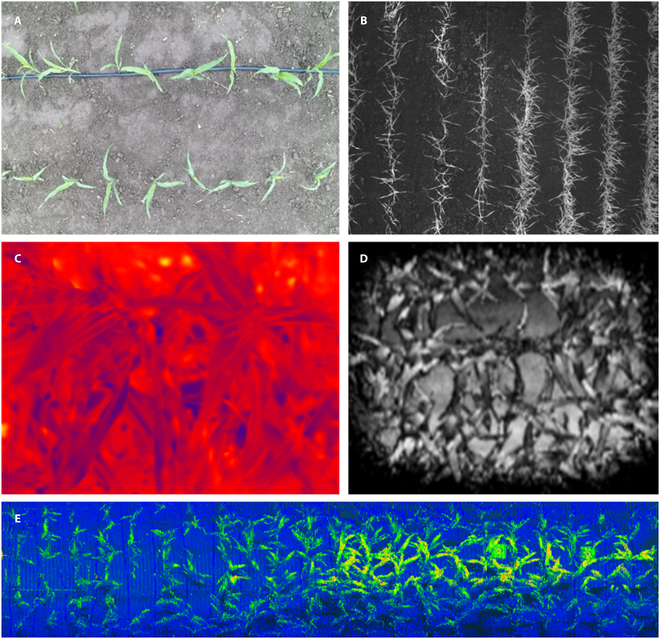
The images acquired by the sensors: (A) RGB image, (B) multispectral image, (C) thermal infrared image, (D) deep image, and (E) 3D lidar point cloud data.

The set route overall efficiency could reach 45 plants/min. According to the measurement system size, it could measure hundreds, thousands or even tens of thousands of plants, or hundreds of communities daily. Suitable plants for measurement included maize, wheat, rice, soybean, vegetables, flowers, fruit trees, and other plant groups. The images acquired by the sensors are shown in Fig. 2.

### Software control system

**Fig. 3.**
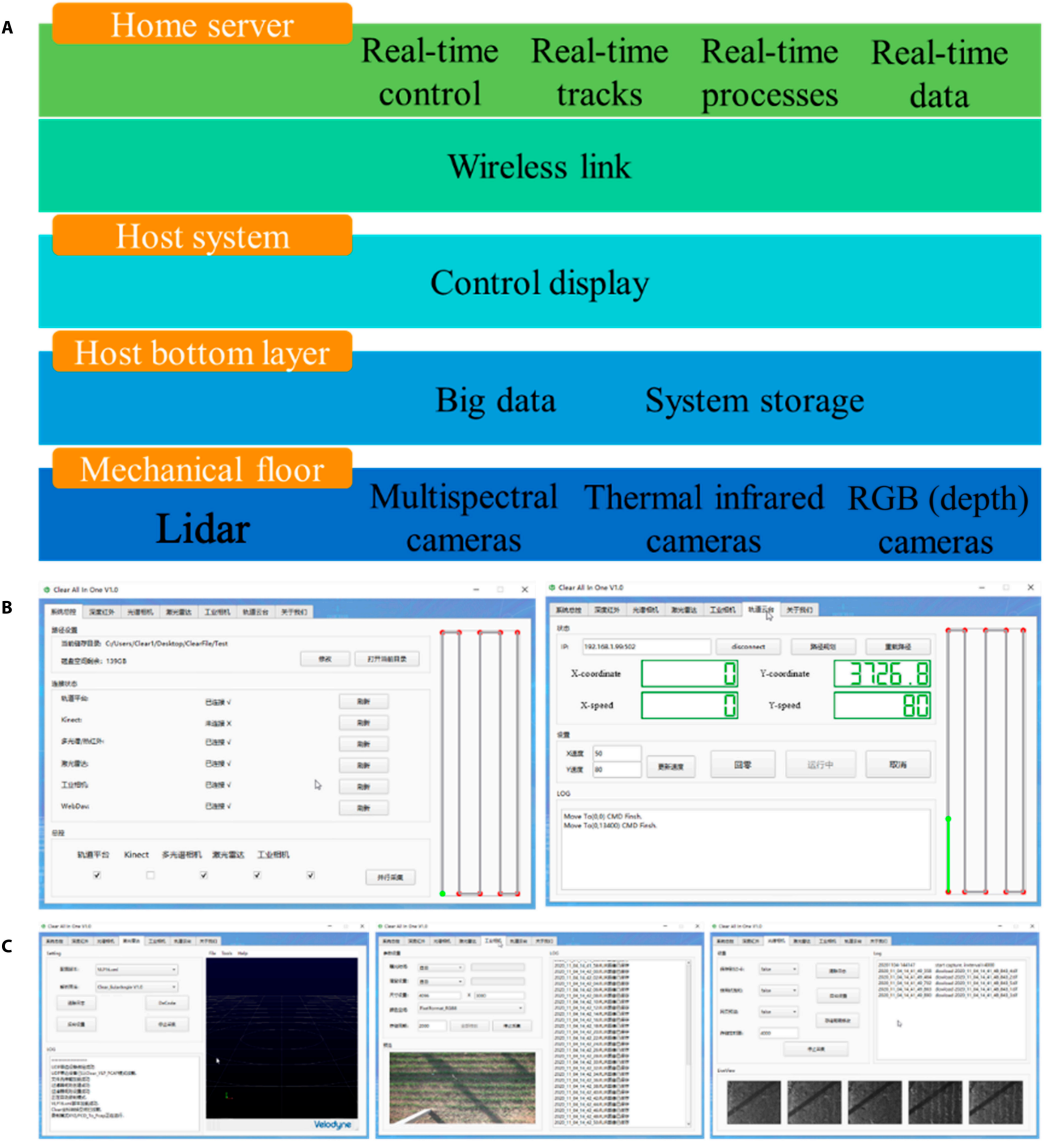
The control system: (A) phenotyping platform control schematic diagram of the remote control system, (B) phenotyping platform remote control system, and (C) interactive interface of the main sensors.

The control diagrams of the phenotyping platform and the remote control system of the phenotyping platform are shown in Fig. 3A and B, respectively. The operating system included a host computer and a local server. The operational interface of the host computer could be logged in a short-distance range via Wi-Fi (within 50 m without shielding) or remotely via LAN. It was used for pre-operation preparation and data acquisition control, which included the processes of experiment registration, platform status monitoring information, control of each sensor and fill light, track path planning, real-time visualization of the data acquisition by a bridge process, data storage, and data transmission. The local server was connected to the host remotely, which could perform real-time job control, status monitoring, data return, data management, and statistical analysis of the job process.

The PLC platform controlled the *XYZ* 3-axis motor to move in the entire study area. The platform PLC control box was set next to the track-type platform. The PLC host was connected to the network with the RJ45 interface and could be used by the NUC industrial control host equipped with professional software to communicate with the host computer. The values of real-time *XY* coordinates and speed were displayed in the LCD number box, and the path was displayed on the right side. The green point denotes the current coordinate point and the grey line represents the running route; the green line indicates the running course. The software could modify the parameters of each of the sensors, such as exposure time and data storage format, according to the specific environmental conditions.

The system main interface is shown in Fig. 3C, where it can be seen that it included the main overview information bar, sensor status bar, deep camera operation bar, multispectral operation bar, and lidar operation bar. The multispectral operation bar included information on the sensor connection status, SD card status, and settings, such as photographing mode, which was used to preview and set the multispectral sensor. The overview bar showed the GPS status, remaining space in the directory tree, and an optional storage folder to store the collected data. The deep camera operation bar included the sensor acquisition speed and the preview of the RGB, depth, and infrared images.

### Phenotyping assessment

In this study, lidar data were used to estimate the phenotyping of maize (includes plant height, leaf length, leaf width, etc.). The specific steps of the data acquisition and processing process were as follows:

• calibrate the lidar to obtain the internal parameter data. After deploying the sensor, select appropriate light and wind speed conditions for data acquisition;
• perform statistical filtering on a single-point cloud data to remove the noise and retain only the maize plants and soil in the filtered point cloud data;
• segment the filtered point cloud data by a random sampling consistency algorithm, fit and remove the soil layer plane, and retain the data on maize plants above the soil layer;
• and use deep learning method to segment multiple plants in a single point cloud and then calculate the phenotyping using previous algorithms we have done [29].

#### Sensor calibration

**Fig. 4.**
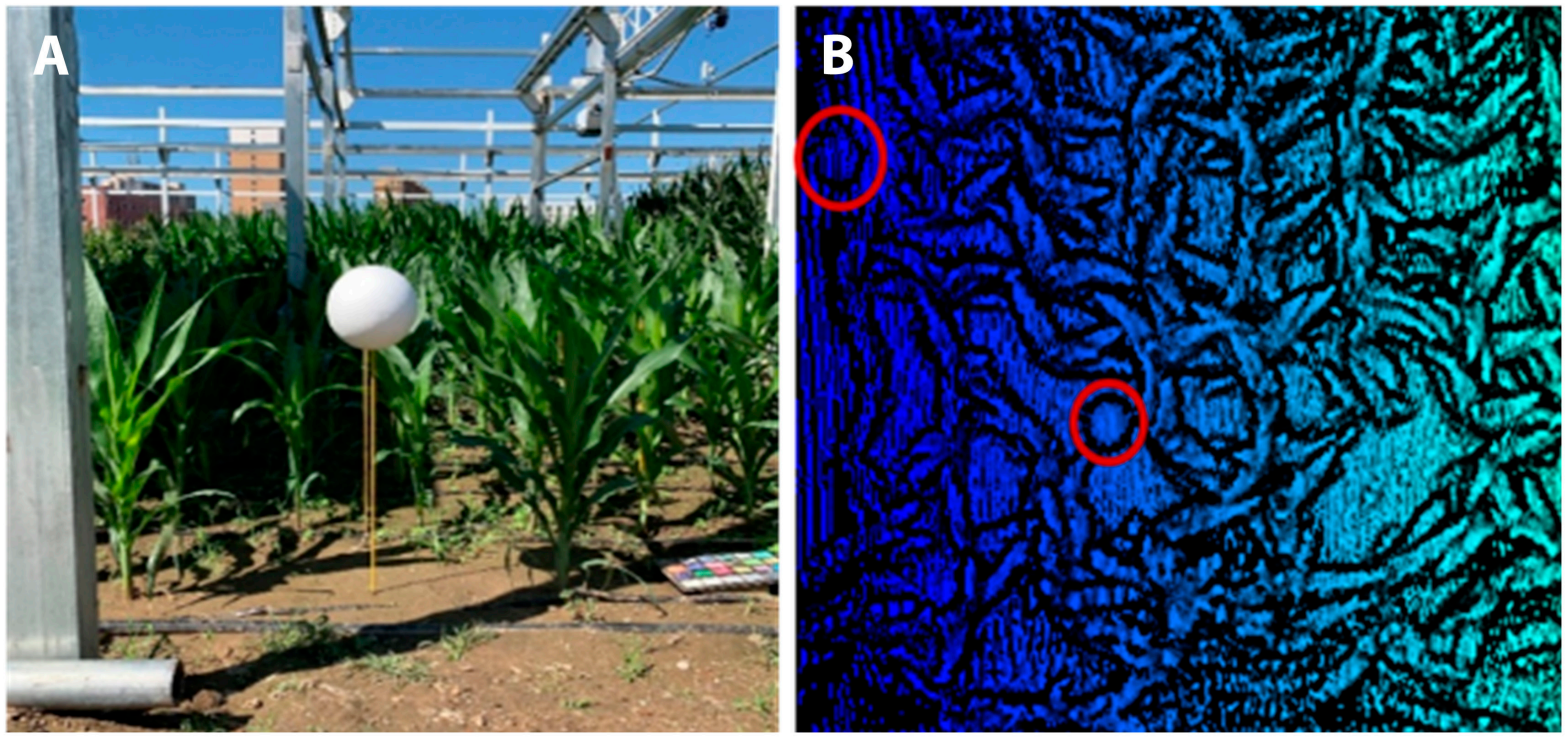
The 3D calibration ball application: (A) picture of the 3D calibration ball in the field and (B) calibration balls in the point cloud data.

Because of the problems in the manufacturing process and structure installation and other 3D space-scanning equipment problems, imaging errors and internal distortion of the 3D scanning equipment were unavoidable [34]. To calculate the internal error parameters of the camera, 3D equipment was required to be recalibrated after a certain period. Two calibration balls with diameters of 250 and 200 mm were placed in the experimental planting area where the blade was not shielded and a fixed-scale point cloud was introduced. The recalibration could increase the accuracy of the subsequent analysis of the point cloud phenotype parameters [35]. The position of the calibration ball in the field and the point cloud data are shown in Fig. 4.

The equipment is calibrated before each run using a calibration toolbox. The solution of the internal and external parameters and aberrations of the camera can be obtained by calibrating the template with the Camera Calibration Toolbox. The corner points of the calibration template can be extracted with the help of this toolbox, and the projection transformation relationships between multiple corner points of each of the *n* different images can be calculated. The 5 internal and 6 external parameters of the camera are then solved by the inner closure method. Finally, the Levenberg–Marquardt algorithm is used to optimize all the parameters for the recalibration of the sensor.

#### Point cloud registration

A collinear equation defines a model with the most rigorous registration between a single image and a 3D point cloud. Still, the collinear equation has strict requirements on the initial values of the internal and external azimuth elements [36,37]. Therefore, in this study, the direct linear transformation (DLT) [38] method was selected to register an orthoimage of the field maize population processed by the Open Drone Map software using the point cloud data obtained by lidar scanning. The DLT method performs the registration by establishing a direct linear relationship between the plane coordinates of critical points in an image and the spatial coordinates of points in the corresponding 3D point cloud data. The DLT is a simplified linear model of the collinear equation, and it can be expressed as follows:

$$x+\frac{l_{1}X+l_{2}Y+l_{3}Z+l_{4}}{l_{9}X+l_{10}Y+l_{11}Z+1}=0$$ (1)

$$y+\frac{l_{5}X+l_{6}Y+l_{7}Z+l_{8}}{l_{9}X+l_{10}Y+l_{11}Z+1}=0$$ (2)

where (*x*, *y*) denotes 2D coordinates of a feature point in an image, (*X*, *Y*, *Z*) represents a characteristic point space coordinate in the 3D point cloud data at a known position, and *l*_1_ to *l*_11_ denote sets of unknown parameters required to calibrate the model.

**Fig. 5.**
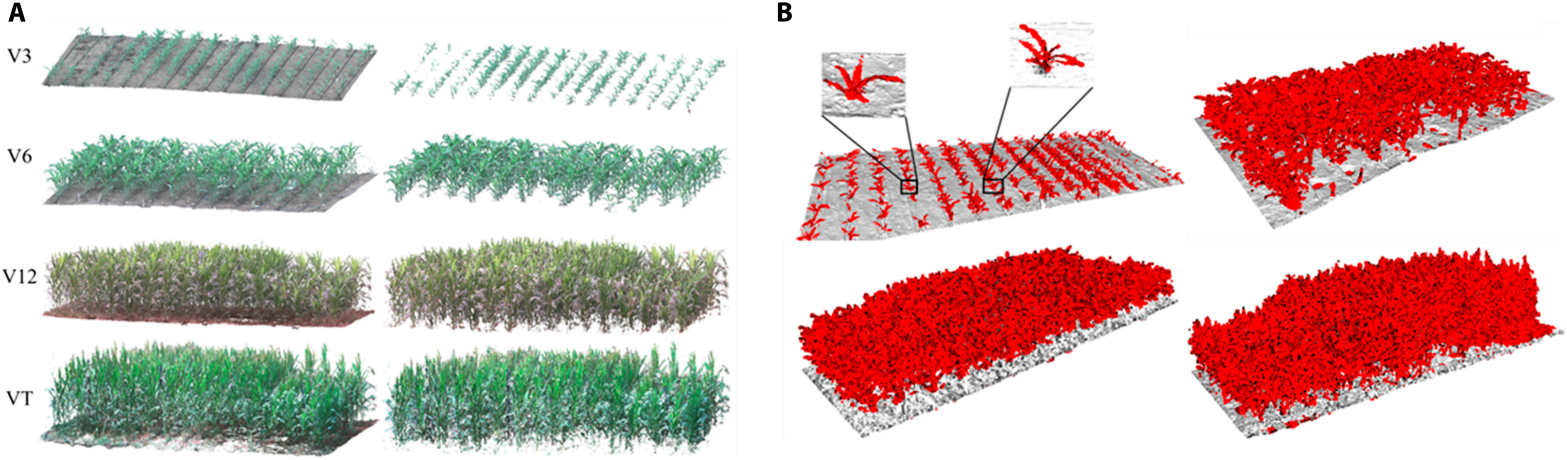
(A) Registration result of the maize population point cloud and the removal effect of the ground point cloud in different growth periods and (B) details of the removal results of ground point clouds.

Figure 5 shows the results of the point cloud registration and ground point cloud removal for the field maize population in the growth period obtained by the proposed method. The upper left maize in Fig. 5B shows the details of removal results of the ground point cloud, where it can be seen that the removal effect of the ground point cloud for the field maize population was relatively ideal when the algorithm was used [39,40].

#### Point cloud filtering

In 3D modeling, to ensure model authenticity and reduce the point cloud hole, the density of the original point cloud data must be high. However, in crop phenotypic analysis, the dense point cloud can reduce the data processing speed of a computer. The purpose of down-sampling is to process the maize population point cloud data by a specific algorithm, replace a plurality of points in the neighborhood with one point, and substantially reduce the number of problems while maintaining plant shape characteristics. The voxel filter can achieve down-sampling without destroying the geometry of the point cloud itself. In addition, the voxel filter can remove a certain degree of noise points and outliers.

**Fig. 6.**
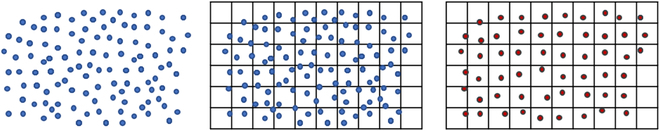
The 2D diagram of the point cloud subsampling by the voxel raster method.

**Fig. 7.**
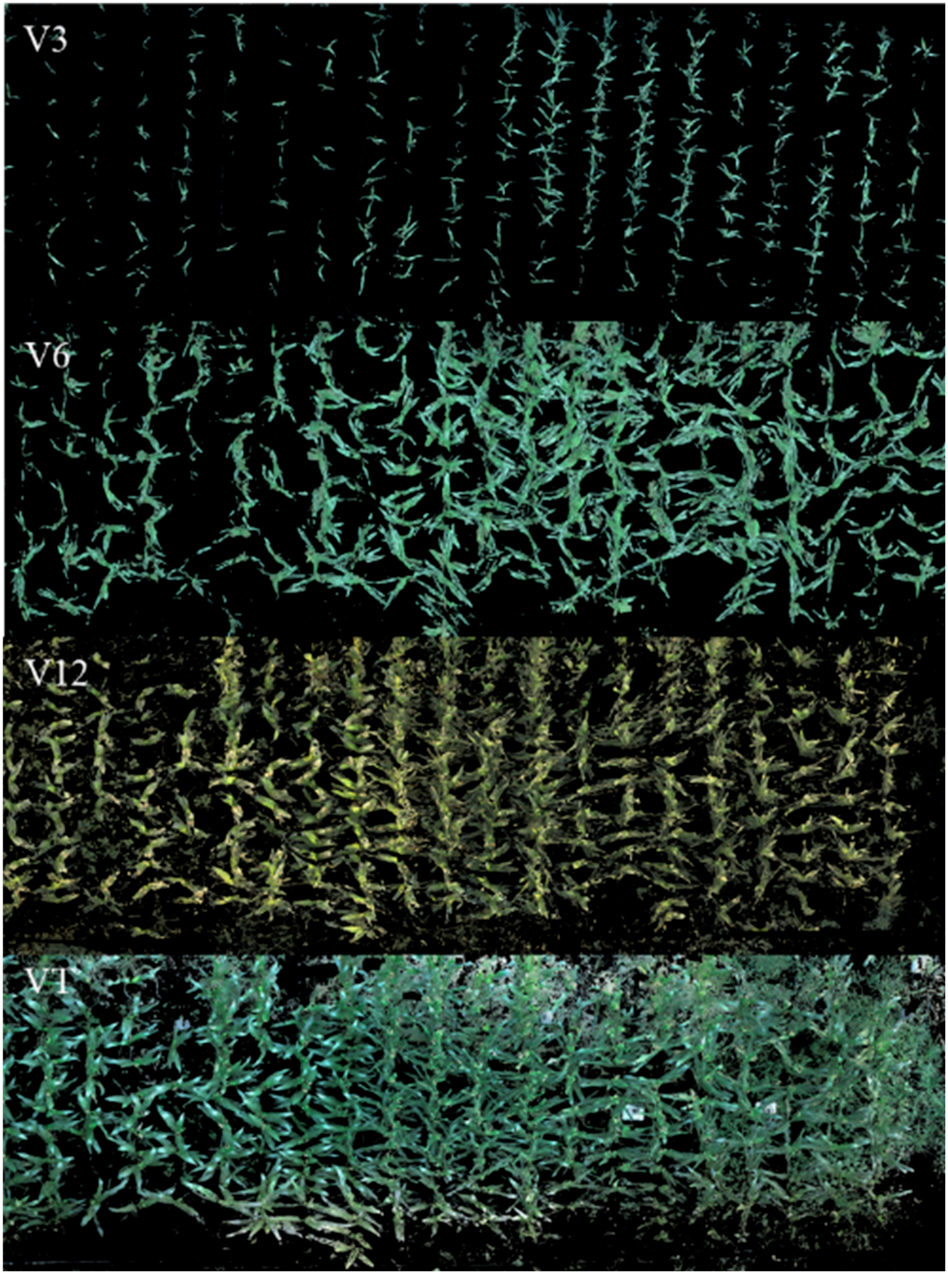
Results of the point cloud data after voxel lattice sampling.

Theoretically, the volume pixel (voxel) is similar to the smallest unit pixel in a 2D space. In the proposed system, a pixel was applied to image data of 2D computer images and denoted a basic unit of image display. It should be noted that a voxel is the most minor unit voxel lattice point subdivided continuously in a 3D space and sampling is to create a 3D voxel lattice based on the obtained original maize population point cloud. A voxel grid reflects or presents plurality of cubes (voxels) in a 3D space. A 3D voxel grid contains all discrete points of a maize cluster point cloud and in each voxel. The point cloud filters data obtained by replacing other points in the voxel with the gravity center of all points. Finally, the point cloud is converted into voxels. The 2D schematic diagram of the point cloud subsampling by a voxel raster method is shown in Fig. 6. In this study, the point cloud density was relatively uniform for the point cloud obtained by lidar scanning. Voxel lattice sampling could reduce the point cloud density without destroying the point cloud characteristics, as shown in Fig. 7.

#### Crop population segmentation

**Fig. 8.**
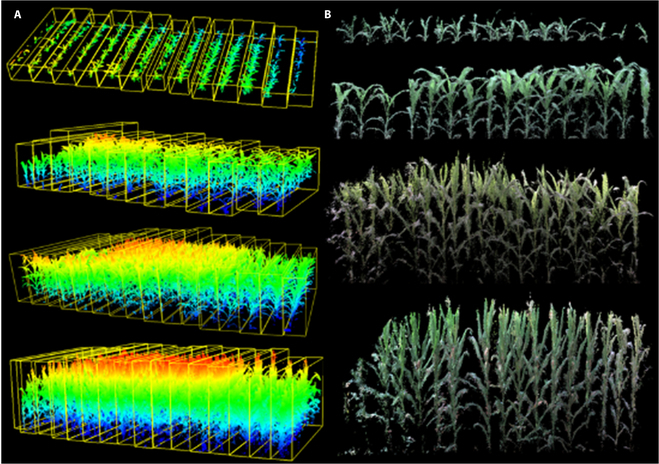
Segmentation result of the maize population point cloud data after voxel slicing: (A) segmentation result of the maize point cloud data after voxel slicing and (B) the side view of an individual plant from the group point cloud presented in (A).

To calculate the phenotyping of the maize plant from the 3D point cloud from the top view better, the soil layer plane and irrelevant objects were removed from the point cloud data, and the complete plant point cloud was extracted from the complex environment background. The scene of the point cloud data from the top view was not complex; there were only a few unrelated objects, such as plants, soil surface, and a drip irrigation pipe, and most of the irrelevant objects were near the soil layer. Therefore, the random sampling consistency method was used to remove the plane fitting of the soil layer, and, finally, complete and clean plant point cloud data were obtained. The segmentation result of the maize population point cloud data after voxel slicing is shown in Fig. 8.

#### Plant phenotyping extraction

**Fig. 9.**
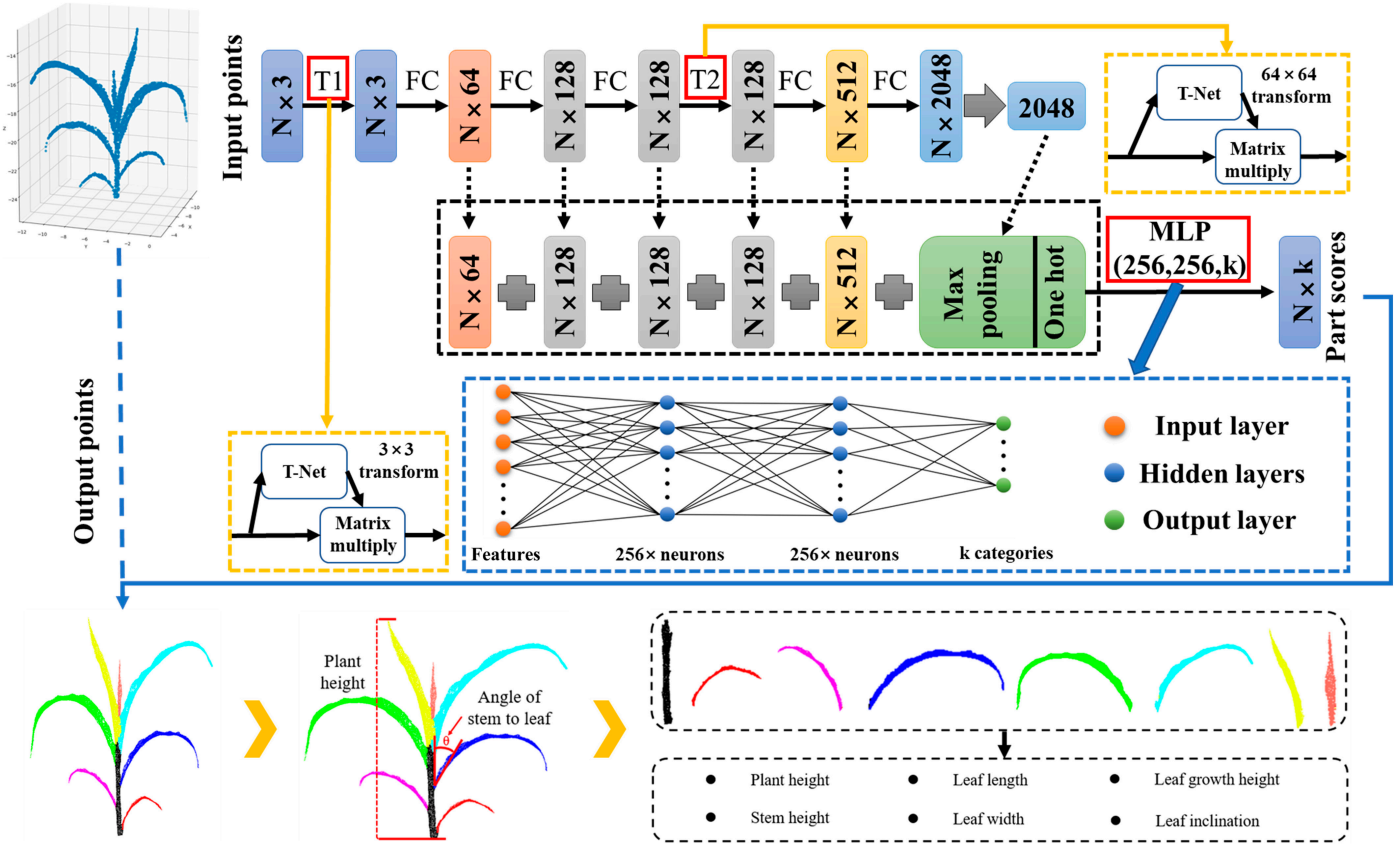
Schematic diagram of the single maize phenotyping measurement.

The point cloud data of the maize plant after segmentation were segmented by the method introduced with Li et al. [29]. Subsequently, several phenotypic parameters were extracted, including plant height, leaf length, leaf width, and leaf inclination. First, the point cloud data of a single maize plant of each variety were obtained, and the straight line distance from the highest point of the maize plant to the ground point was calculated; this distance represented the plant height. In Fig. 9, the *Z* axis corresponds to the vertical growth direction of the maize plant, *Z*_max_ is the highest point of the maize growth, *Z*_min_ is the lowest point of the maize growth, and height represents the plant height of a single maize plant; the plant height denoted the difference between *Z*_max_ and *Z*_min_. Leaf length and leaf width were calculated using the leaf segmented point clouds. Meanwhile, the leaf inclination was obtained by calculating the leaf stem angle θ as a complementary of the stem–leaf angle. The specific phenotypic parameters extracted are shown schematically in Fig. 9.

The network shown in Fig. 9 was trained with 500 EPOCHs. The total time for model training was approximately 54 h on a workstation with 2 Intel Xeon (R) Gold 6248 CPUs, 256 GB of RAM, and 2 NVIDIA Quadro RTX6000 GPUs. The Softmax cross-entropy function was used as a loss function during training and defined as follows:

$$\text{Loss}=\sum_{n=1}\left(-y_{n}\times \log\left({\hat{y}}_{n}\right)\right)+L_{\mathrm{reg}}$$ (3)

where *n* is the total number of points in the input point cloud, *y_n_* is the ground truth of the multilevel classification corresponding to this point cloud, and ${\hat{y}}_{n}$ is the probability of the output of each point cloud category using the Softmax function. More detailed parameters about this model can be found in the work of Li et al. [29].

### Evaluation metrics

The plant height data extracted from the point cloud data were compared with the manual measurement results. In this study, the manual measurement results were obtained by the experimenters in the field. Namely, the daily plant height measurement data of all plants in the study area were averaged, and the average value was used as a representative value of the plant height for that day. The correlation coefficient (*R*^2^) and the root mean square error (RMSE) were selected to assess the results, and they were calculated as follows, respectively:

$$R^{2}=1-\frac{\sum_{l=1}^{m}{\left(v_{l}-v_{l}^{\prime }\right)}^{2}}{\sum_{l=1}^{m}{\left(v_{l}-{\bar{v}}_{l}\right)}^{2}}$$ (4)

$$\text{RMSE}=\sqrt{\frac{1}{m}\sum_{l=1}^{m}{\left(v_{l}-v_{l}^{\prime }\right)}^{2}}$$ (5)

where *m* denotes the number of comparison objects, $v_{l}$ is the manual measurement result, $v_{l}^{\prime }$ is the phenotypic parameter extracted from the automatic segmentation result using the algorithm, and ${\bar{v}}_{l}$ is the mean value of the manual measurement result.

## Results

### Optimal sensor height verification

**Fig. 10.**
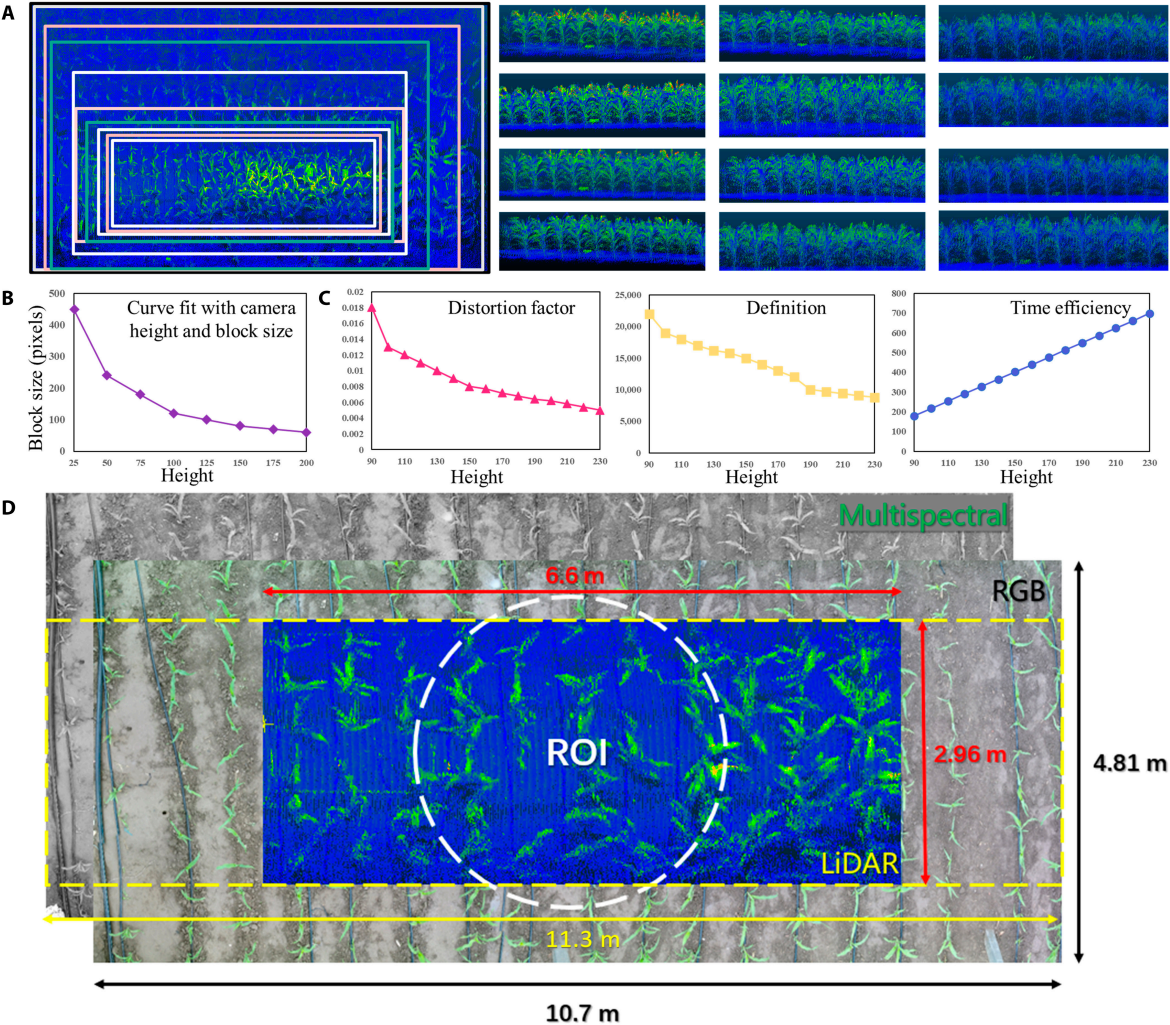
Optimal height values of sensors and phenotyping platform: (A) 3D lidar, (B) multispectral camera, (C) RGB camera, and (D) the central image unit of the phenotyping platform. ROI, region of interest.

Before collecting the data, the best field angle, and height of the lidar, multispectral camera and RGB camera were evaluated, and the results are shown in Fig. 10. For lidar, from top to bottom and from left to right, the optimal height values were as follows: 100, 110, 120, 130, 140, 160, 170, 180, 190, 200, 210, and 230 cm. On the basis of the texture data of the point cloud, the sensor needed to be far away from the canopy to obtain a large angle of view. The lower the distance from the canopy was, the clearer the texture details of a plant were, as shown in Fig. 10A. Multispectral images were acquired at different heights, and 5 sensor-matching parameters were calculated for the multispectral camera. First, the translation parameters of the 5 sensor images were calculated at 5 height values (30, 55, 105, 155, and 205 cm), and the *x* and *y* translation parameters were fitted to obtain the translation parameters at any given height. Then, an image pixel resolution at different image heights was calculated, and the pixel size of the imaged object in the image at any height was fitted, as shown in Fig. 10B. For the RGB camera, 4 indexes of distortion coefficient, definition, time efficiency, and data redundancy were considered the compatibility of the field of view of each sensor, and the current requirement of the extracted parameter information were analyzed; the optimal shooting height of the image was obtained to be 1.7 to 1,9 m, as shown in Fig. 10C.

As shown in Fig. 10D, the central imaging unit had the most significant utility ratio at a distance of 1.8 m from the crop canopy and could obtain accurate RGB, multispectral, and lidar data in terms of plant details and acquisition efficiency. The size of the positive mosaic RGB and multispectral images was 10.7 m × 4.81 m, and the lidar data area was calculated considering the optimal field of view angle and operating speed to be 6.6 m × 2.96 m. The overlapping area of the 3 sensors was regarded as a region of interest, and the region of interest was constantly determined to calculate and analyze the plant phenotypic indicators.

### LQ-FieldPheno facility application to breeding plots

**Fig. 11.**
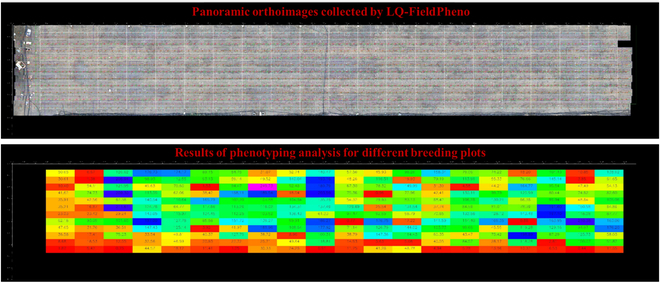
Automatic stitching results of panoramic orthophotos of large fields (top) and automatic segmentation results of planting plots (bottom).

The upper left corner of the field crop phenotyping high-throughput platform was set as the origin of the coordinate system. The sequential orthophoto images acquired by the field phenotyping platform and the coordinates of each photo were processed in batches. Automatic stitching of the global orthophotos was performed by a homonymous image point matching algorithm. An international orthophoto image was obtained for the area covered by the device, as shown in Fig. 11. This image was used for the automated analysis of field phenotypes conducted by the image processing algorithms and for a quick check of the completeness of the collected data.

Next, each planting plot was segmented and labeled according to the initial planting map of the field and the centroid of each maize plant; the level set algorithm was used to extract the maize centroid. Then, the collated plots were used to obtain the attribute information of each material. The phenotypic parameters obtained using a high-throughput analysis algorithm were used to compare differences in parameters between varieties, for instance, critical phenotypic traits, such as plant height, cover, leaf area index, and biomass.

### Maize point cloud data preprocessing analysis

**Fig. 12.**
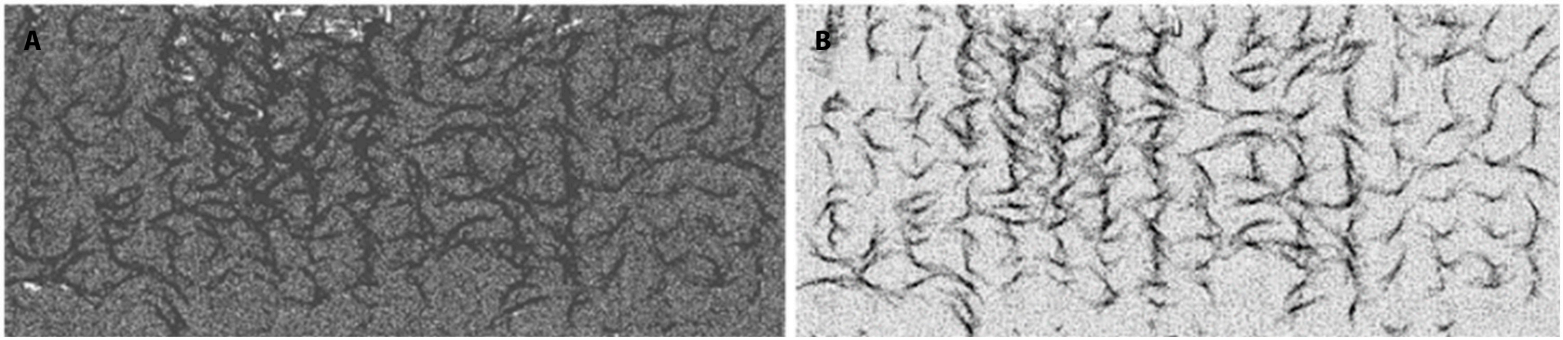
The maize population data before and after the point cloud subsampling: (A) before the point cloud subsampling and (B) after the point cloud subsampling.

For data with a relatively large number of lidar point clouds, but relatively uniform point cloud density, the voxel grid point method can be employed for a down-sampling operation to reduce the number of point clouds, which is essential for achieving the real-time performance of point cloud filtering. The data before and after the point cloud down-sampling are shown in Fig. 12A and B, respectively, where it can be seen that, after the point cloud down-sampling, the morphological characteristics of maize were unchanged, but the point cloud density was substantially reduced.

**Fig. 13.**
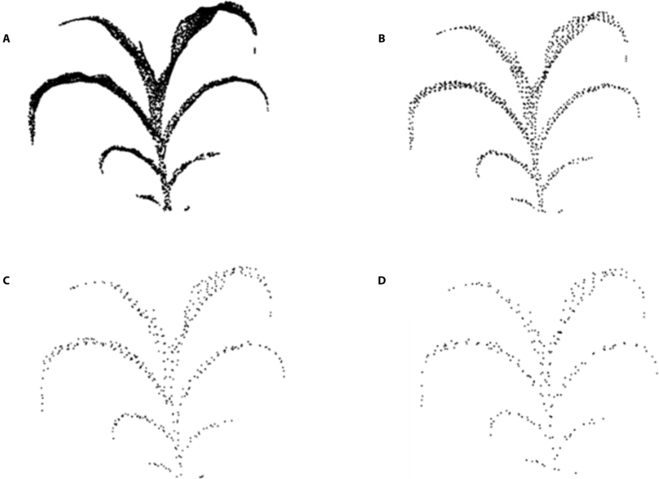
Subsampling results of a single maize point cloud data for different grids: (A) the original point cloud, (B) 0.2-cm grid, (C) 0.5-cm grid, and (D) 1-cm grid.

When the 3D voxel grid was a cube with a side length of 1 cm, the number of points before down-sampling was 14,685,679 and the number of points after filtering was 521,477, so the compression rate was 36%. The more lidar scanning lines were in the equipment selection, the more point clouds were. Therefore, the point clouds generated by the lidar and depth cameras were selected one by one by adjusting the side length of the voxel grid according to actual requirements. According to the volume size of the directional bounding box and the number of point clouds in a unit volume, the values of 0.2, 0.5, and 1 cm were selected as grid side lengths to perform point cloud down-sampling. To evaluate the down-sampling effect, a single maize plant data were processed for different grids, and the algorithm processing effect is shown in Fig. 13.

The experimental results show that, when the grid edge length was less than 0.1 cm, the result was close to the original point cloud data, but the sampling efficiency was low. However, when the grid edge length was longer than 1 cm, the outline shape of the point cloud data before and after the sampling was unchanged, saving many computing resources in subsequent operations. The point cloud data reading time results of the lidar before and after the point cloud down-sampling are shown in Table 2.

**Table 2.** Comparison results of the file reading time of the lidar before and after point cloud simplification.

Processing	Grid length grid	File reading time
Before subsampling	-	121
After subsampling	0.2	35
	0.5	18
	1	7

### Maize phenotyping calculation and analysis

The maize plant height is an essential indicator of maize phenotypic parameters, and it is closely related to the lodging resistance of maize stalks. After correction by the point cloud coordinate system, the ground slope was reduced. The ground 3D point cloud fluctuated less in the *z* axis direction than *x* and *y* axes. At the same time, the plant height of a single maize plant was calculated as a distance from the highest point of the natural growth of maize to the ground.

**Fig. 14.**
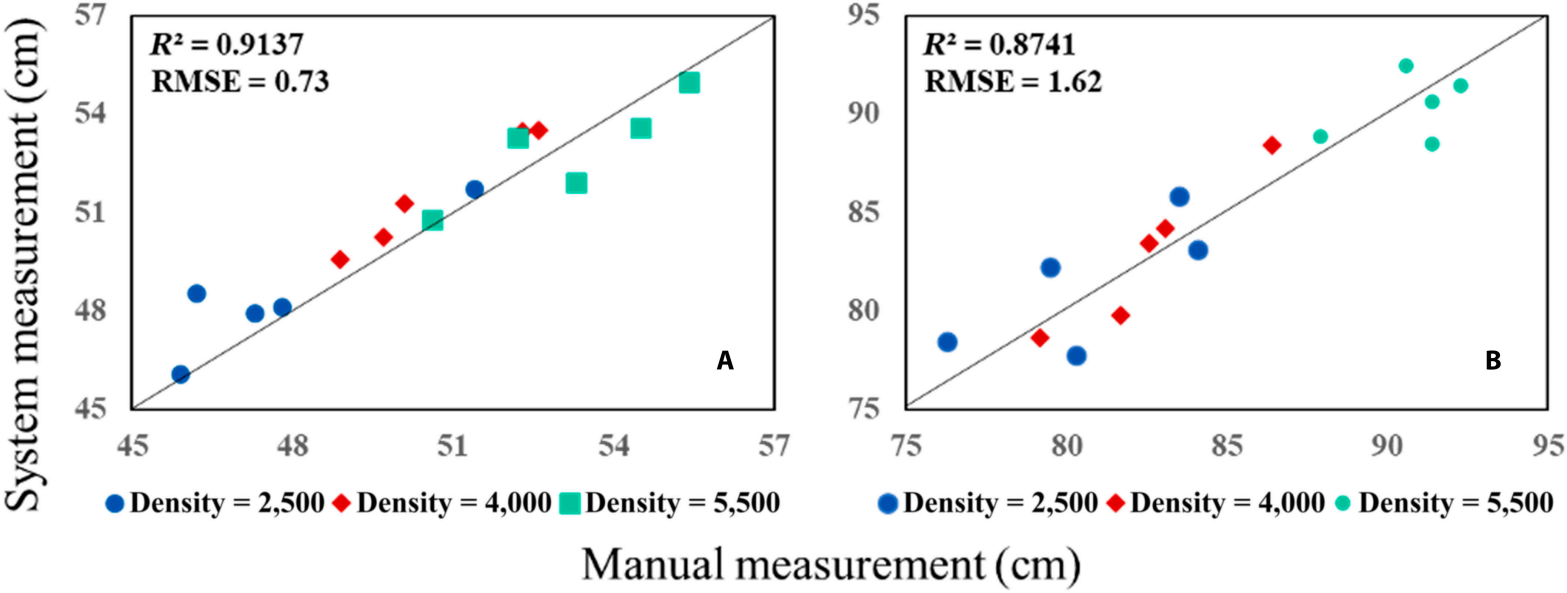
Regression analysis results of the maize plant height obtained from the lidar data: (A) 2020 August 14 and (B) 2020 August 27.

In the error analysis of the plant height data obtained by the 3D lidar, the plant heights corresponding to 3 density values of AD268 maize varieties of 2,500, 4,000, and 5,500 plants per area on 2020 August 14 and 27 were selected. The comparison results of the systematically and manually measured values of the plant height on the 2 d are given in Table 3. As shown in Table 3, on 2020 August 14, the average relative errors of the 3 densities were less than 2%, and on 2020 August 27, the maximum and minimum relative errors were 3.45% and 0.71%, respectively. The RMSE and *R*^2^ results on 2020 August 14 and 27 were 0.99 cm and 0.9137, as well as 1.80 cm and 0.8471, respectively. In addition, the achieved linear fitting effect was good. The regression analysis results of the maize plant height are shown in Fig. 14.

**Table 3.** Comparison of manual and systematic measurement results of the maize plant height obtained from the lidar point cloud data.

Date	Variety	No.	Manned measurement (cm)	Systematical measurement (cm)	Error (cm)
2020 August 14	AD268	1	47.3	47.96	0.66
		2	45.9	46.1	0.2
		3	47.8	48.13	0.33
		4	51.4	51.71	0.31
		5	46.2	48.56	2.36
	Average	-	47.72	48.492	0.772
	AD268	1	52.6	53.49	0.89
		2	52.3	53.47	1.17
		3	49.7	50.27	0.57
		4	50.1	51.29	1.19
		5	48.9	49.57	0.67
	Average	-	50.72	51.618	0.898
	AD268	1	52.2	53.26	1.06
		2	50.6	50.79	0.19
		3	53.3	51.91	1.39
		4	54.5	53.6	0.9
		5	55.4	54.98	0.42
	Average	-	53.2	52.908	0.792
2020 August 27	AD268	1	76.3	78.48	2.18
		2	79.5	82.24	2.74
		3	80.3	77.79	2.51
		4	84.1	83.15	0.95
		5	83.5	85.81	2.31
	Average	-	80.74	81.494	2.138
	AD268	1	79.2	78.64	0.56
		2	82.6	83.41	0.81
		3	83.1	84.19	1.09
		4	86.4	88.41	2.01
		5	81.7	79.76	1.94
	Average	-	82.6	82.882	1.282
	AD268	1	87.9	88.83	0.93
		2	91.4	90.6	0.8
		3	91.4	88.51	2.89
		4	90.6	92.43	1.83
		5	92.3	91.46	0.84
	Average	-	90.72	90.366	1.458

**Fig. 15.**
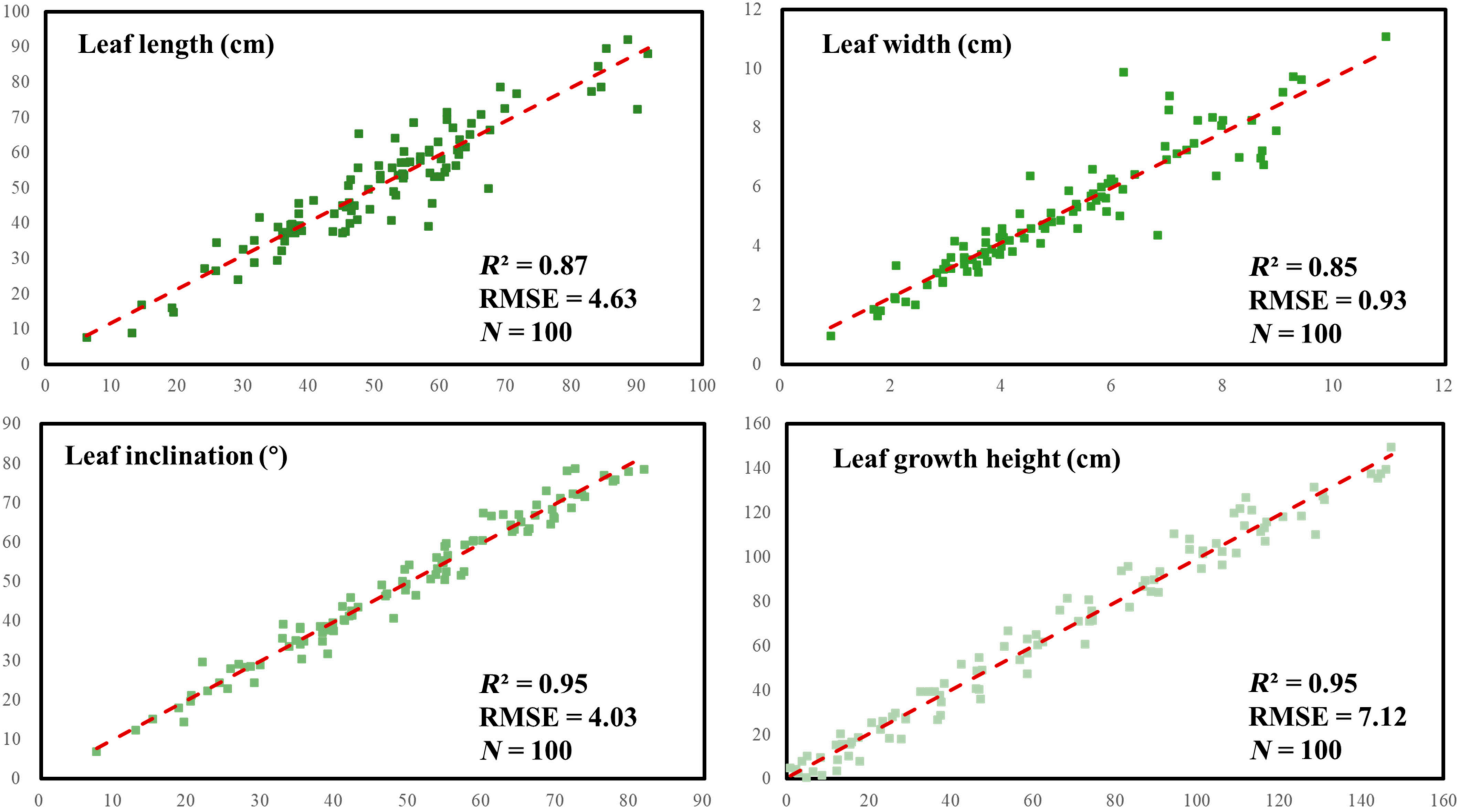
Regression analysis results of the maize plant phenotypic parameters obtained from the lidar data.

The validation results for the other phenotypic parameter are shown in Fig. 15. In the comparative results for leaf length, leaf width, leaf inclination, and leaf growth height, *R*^2^ and RMSE were 0.87 and 4.63 cm (leaf length), 0.85 and 0.93 cm (leaf width), 0.95° and 4.08° (leaf inclination), and 0.95 and 7.12 cm (leaf growth height), respectively. The correlation between the extracted results for leaf width was low, and the correlation for leaf length was medium, while the correlation for leaf inclination and leaf growth height was high.

## Discussion

Many factors can cause phenotypic measurement errors in the test process. First, when a fixed phenotypic platform is used to extract in situ phenotypic parameters of a maize plant in the field, disturbances in light, wind, and other natural factors can reduce the number of points at the blade edge substantially. The hole in the leaf point cloud data can result in inaccurate calculation results of the highest point of a plant and, thus, affects the calculation precision of plant height measurement. In addition, it is difficult to cluster and count the plant leaves and extract the skeleton.

Furthermore, under the planting density of 4,000 plants per area, there is a leaf shading problem between maize varieties after the 9-leaf period. Among the maize types with noticeable phenotypic differences in plant height, the highest point of the dwarf-type plant was blocked fast by leaves of other varieties, resulting in a lack of plant height information on certain groups. In addition, the higher the plant is, the more difficult it is for a leaf closer to the ground to be captured by a 3D scanning device. When the leaf shade is severe, it is challenging to segment a single maize plant, and there is also a problem with the ownership determination of a leaf, which makes it difficult to determine the plant to which the leaf belongs; thus, it is impossible to cluster leaves accurately.

To solve the abovementioned problems, in future experiments, data acquisition could be conducted in the morning or evening hours when the light is not intense and wind interference can be avoided. For the plant phenotypic measurement performed after the maturity period, the planting density should be reduced, or the plant and row spacing should be increased under specific test conditions. In this way, the measurement accuracy could be improved to a certain extent, ensuring a better phenotypic analysis effect.

Considering that electricity is the power source for the whole platform at this stage, an interesting direction for our future research is to find new sources of energy to replace electricity in order to save energy and reduce emissions. Solar energy might be a good option, and there are already many successful examples of greenhouse solar farms. The solar production system has been widely presented in a number of related studies [41–43]. It could be considered for integration into plant phenotyping platforms to achieve energy savings.

### Conclusions

This paper presents an integrated phenotypic measurement platform based on a near-surface remote sensing technology, which can effectively address the existing phenotypic data collection problems. The proposed platform provides a powerful research method for plant biology, phenomics, precision cultivation management, and breeding. However, the throughput of genotype and phenotype data can create significant data in the breeding science. In addition, for high-throughput data, there are problems with the storage, management, and analysis of massive data. Therefore, the phenotype measurement platform should be further improved and innovated, and its application fields should be expanded.

Future application and development of the proposed integrated platform should focus on the following 3 aspects:

1. Improvement in the integration and stability of the system platform to promote its application in large-scale industrial breeding. In terms of hardware, the interface connection and data transmission of various types of sensors could be considered, and the possibility of integrating more types of sensors into the platform could be analyzed.
2. Software that can meet the requirements of efficient data analysis under the condition of big data should be developed. In addition, to improve the efficiency of data acquisition and processing, software and hardware should be integrated to enhance their efficiency.
3. By adopting interdisciplinary cooperation, the phenotypic, environmental, and genotype data could be analyzed to explore the interaction mechanism of the genotype, phenotype, and environment. At the same time, the massive data could be managed and analyzed to transform the high-throughput phenotypic parameters into model parameters, establish accurate crop growth models, and validate virtual models as metadata. It could be expected that, in the near future, because of the development of sensors and the progress of computer science, a platform for crop phenotypic measurement would be able to obtain more accurate multidimensional phenotypic parameters, ushering in a new era of phenomics and breeding.

## Data Availability

The data could be given upon reasonable request from the corresponding authors.
